# Wide intra- and inter-country variability in drug use and dosage in very-low-birth-weight newborns with severe infections

**DOI:** 10.1007/s00228-012-1415-2

**Published:** 2012-10-05

**Authors:** Chiara Pandolfini, Florentia Kaguelidou, Marco Sequi, Evelyne Jacqz-Aigrain, Imti Choonara, Mark A. Turner, Paolo Manzoni, Maurizio Bonati

**Affiliations:** 1 Laboratory for Mother and Child Health, Department of Public Health “Mario Negri” Pharmacological Research Institute, Via Giuseppe la Masa 19, 20156 Milan, Italy; 2 Department of Pediatric Pharmacology and Pharmacogenetics, Hôpital Robert Debré, AP-HP; INSERM CIC9202, University Paris 7 Diderot, Paris, France; 3 Academic Division of Child Health, University of Nottingham Derbyshire Children’s Hospital, Uttoxeter Road, Derby, DE22 3DT UK; 4 Department of Women’s and Children’s Health, Institute of Translational Medicine, University of Liverpool–Liverpool Women’s Hospital, Crown Street, Liverpool, L8 7SS UK; 5 Neonatology and Hospital Neonatal Intensive Care Unit, Azienda Ospedaliera Regina Margherita–Sant’Anna, Corso Spezia 60, 10126 Turin, Italy

**Keywords:** Data collection, Intensive care units, neonatal, Sepsis, Ciprofloxacin, Fluconazole

## Abstract

**Purpose:**

To describe the use of ciprofloxacin and fluconazole for the treatment of sepsis in European neonatal intensive care units (NICUs) in order to better orient research aimed at acquiring essential knowledge in this critical area.

**Methods:**

The survey consisted of an online questionnaire for all participating NICUs on treatment schemes employed, rationales behind drug choices and interest in participation in research involving the two drugs.

**Results:**

A total of 189 level II and III NICUs participated in the survey, representing 25 countries, with Italy, UK and France providing the greatest number of centres (54 % of total). Ciprofloxacin is used in 25 % of NICUs that responded, although the indications for administering it vary between centres and the dosage ranges vary considerably, with 25 % of NICUs giving ≤10 mg/kg/day and another 25 % giving ≥21 mg/kg/day. Factors given as affecting the decision to use ciprofloxacin are uncertainty about its safety and pharmacokinetics and level of penetration in the cerebrospinal fluid. Among the 70 % of responding units that use fluconazole to treat fungal infection, 45 % administer 6 mg/kg unit doses while 33 % administer 12 mg/kg; 41 % of NICUs use a 24-h interval between administrations while 20 % wait 72 h. Among the responding NICUs, 57 % were willing to participate in a project on ciprofloxacin and 59 % would consider participating in a randomized controlled trial evaluating fluconazole versus micafungin.

**Conclusions:**

Great variability in therapies exists within and between countries. Numerous centres are interested in participating in research on these drugs, highlighting the need for further knowledge on sepsis treatment and European centres’ interest in off-patent medicine research.

## Introduction

Infections in neonates may be caused by bacteria or fungi and may be responsible for neonatal death or short- and long-term sequelae [1–3]. In general, data show that the risk of late-onset sepsis increases with decreasing birth weight and gestational age and that hospital-acquired infections are common among very-low-birth weight infants (VLBW; <1500 g at birth). There is a lack of safety and efficacy data on the use of antibiotics and antifungals in preterm newborns and, consequently, adequate, updated information on the treatment of sepsis is warranted [4, 5]. Treatment of these infections is essential but entails increased risks of death, as with prolonged routine empirical antibiotic therapy, and of possible adverse reactions and the emergence of drug resistance [6–9].

Ciprofloxacin is used to treat sepsis caused by multiple resistant organisms [10]. It is not considered in current guidelines for neonatal sepsis, but is increasingly being used, especially when severe infections are caused by *Enterobacter spp*. resistant to standard treatment and when there is a major risk of cerebral abscess [11, 12]. Fluconazole, on the other hand, is an antifungal that is considered by the Infectious Disease Society of America (IDSA) guidelines to be a reasonable option for treatment of invasive candidiasis, a common and often fatal condition in preterm VLBW infants [13]. There is insufficient data, however, on the pharmacokinetics, efficacy and safety of these two drugs in neonates to permit safe and effective use [11, 12, 14–16]. Moreover, the lack of therapeutic knowledge and suitable formulations for children and the consequent increase in risks associated with off-label drug use are widely acknowledged and [17] particularly critical in newborns [18].

It is in this context that a European project called Treat Infections in Neonates (TINN) was set up (www.tinn-project.org) under the 7th Framework Programme [18], linking 16 partners from seven EU member states. It will evaluate the utility and safety of ciprofloxacin and fluconazole because these two drugs are included in the European Medicines Agency’s priority list of off-patent products with the highest need for studies in preterm and term neonates [19]. The use of these drugs, administered as formulations adapted to preterm and term neonates, will be assessed in order to apply for a Paediatric-Use Marketing Authorization and to establish optimal use of these drugs and guidelines to be used throughout Europe.

A survey was set up as a preliminary part of the TINN project to describe the current use of these drugs in neonatal intensive care units (NICUs) in Europe.

## Methods

The survey, in the form of an online questionnaire with a selection of obligatory items, consisted of four sections aimed at collecting data on each participating NICU in terms of: (1) general information, (2) use of ciprofloxacin and fluconazole, (3) choice to use these drugs or not and the factors influencing the choice and (4) presence in clinical research. The items (Appendix 1) were measured on a 5-point Likert scale (1 = least important, 5 = most important). The questionnaire was partly based on a previous U.S. survey [20], which was also used in a later study administered to individual physicians [21], and has been also used in the TINN’s survey on the prophylactic use of fluconazole [22], as was the Likert scale methodology. This latter consisted in, for each drug, grouping respondents into those who used it and those who did not, and then comparing their responses after dichotomization. The complete methodology has already been described in these articles. Categorical variables were compared by χ^2^ analysis and the *P* values are two-tailed. Data were managed using Microsoft Access and analysed using SAS ver. 9.12(SAS Institute, Cary, NC).

From December 2009 to May 2010 various methods were used to contact the greatest number of NICUs in order to be as representative as possible of the European situation (27 member states, plus Turkey and Croatia). Individual researchers, physicians, other healthcare workers and colleagues were contacted, and the Internet and bibliographic databases were searched for additional contacts, including relevant networks and societies. All contacts were emailed an invitation letter to contribute to the survey, asking for participation by a structured staff neonatologist in the NICU and specifying that all participants would be sent a data report. In an attempt to avoid a greater participation of NICUs that use either of the two drugs than of those that do not, the invitation letter did not mention the drugs under study. Additional emails were sent to solicit participation and to complete missing data.

## Results

The data set consisted of completed records from 199 NICUs. In order to have a more homogenous sample of centres, only level II and III NICUs were evaluated. In all, completed questionnaires from 189 level II and III NICUs (25 and 164, respectively) were analysed.

Twenty-five European countries were represented by the data, with the greatest number of participating NICUs located in Italy (38 NICUs), UK (36), and France (28), followed by Sweden (17), Spain (13), Germany (12), Belgium (11), Finland (5), Austria, Hungary, the Netherlands (3 each), Poland, Portugal, Slovakia, Estonia, Croatia, Turkey (2 each), and Luxembourg, Greece, Romania, Slovenia, Denmark, Czech Republic, Bulgaria, Ireland (1 each). Among the participating NICUs, 84 % reported >120 annual admissions and 70 % had between 50–200 annual admissions of newborns at <32 weeks gestational age (22 % had <50 and 8 % had >200). The reported prevalence of fungal infections was low: in 64 % of NICUs it was <1 % and in 94 % <5 %. Regarding bacterial sepsis, the majority of NICUs (72 %) reported a monthly rate of one to ten cases.

### Ciprofloxacin treatment

Most NICUs (89 %) have a standard written protocol regarding antibiotic treatment in cases of suspected bacterial sepsis. Ciprofloxacin, however, is only used in 47 NICUs (25 %). Sweden, with 17 participating NICUs, stands out because none of its NICUs use the drug. Ciprofloxacin is most commonly used in cases of culture-proven bacterial sepsis due to multi-drug resistant organisms that are sensitive to ciprofloxacin (38/47 NICUs). Of these centres, 25 also specified that they used it as first-line therapy.

Seventeen NICUs reported administering ciprofloxacin in cases of severe sepsis resistant to a first-line empirical antibiotic therapy that was not ciprofloxacin. Nine NICUs did not specify the dosage, and the remaining 38 administer ciprofloxacin in dosages that vary enormously both between countries and between NICUs at the national level (Table 1), with the most commonly used regimen being 20 mg/kg/day (42 % of units); however, 27 % of units administer  ≤10 and 19 % administer ≥30 mg/kg/day.

**Table 1:** Average dose of ciprofloxacin used in 38 neonatal intensive care units

Country	Average dose of ciprofloxacin (mg/kg/day)							Total
	<10	10	11–15	20	21–25	30	45	
France		1		6	1	5		13
Italy		2		3				5
Belgium			1	1			1	3
UK	3							3
Poland		1	1					2
Spain				2				2
Turkey		1		1				2
Croatia						1		1
Estonia				1				1
Finland	1							1
Germany				1				1
Hungary			1					1
Portugal					1			1
Romania				1				1
Slovakia		1						1
Total	4 (11 %)	6 (16 %)	3 (8 %)	16 (42 %)	2 (5 %)	6 (16 %)	1 (3 %)	38

Data are presented as the number of neonatal intensive care units (NICUs). Data from 9 NICUs that did not specify dosage are excluded

The main concerns expressed by most NICUs were antibiotic resistance and lack of safety and efficacy data for ciprofloxacin. However, the NICUs that did not use the drug were significantly more likely to be concerned about the scarce information available on the drug’s safety (*p* < 0.01) and kinetics (*p* < 0.03). Ciprofloxacin penetration in the cerebrospinal fluid, on the other hand, was a significant factor in the choice to use the drug (*p* ≪ 0.01).

### Fluconazole treatment

Of all the participating NICUs, 68 % have a standard written protocol regarding fluconazole use in treatment, and 70 % of NICUs administer fluconazole for treatment of systemic fungal infection. Interestingly, 27 % of the NICUs who administer fluconazole do not have a standard written protocol. The dosages used in the different NICUs vary significantly (Table 2), with wide ranges in the unit doses (45 % of NICUs administer 6 mg/kg while 33 % administer 12 mg/kg) and in the interval between administrations (41 % of NICUs follow a 24-h interval while 19 % wait 72 h). When transformed into daily doses, the reported data range from 1 to 20 mg/kg/day, with 34 % of NICUs administering ≤4 mg/kg/day and 49 % administering ≥6 mg/kg/day. Only 16 % of the NICUs administer 12 mg/kg/day, as recommended in the IDSA guidelines.

**Table 2:** Average dosages of fluconazole used in 126 NICUs

Interval (h):	24	24	24	24	48	48	48	72	72	72	Other^a^	Total
Average unit dose:	3	6	12	20	3	6	12	3	6	12		
Country												
Austria			2									2
Belgium		2	1				2			2	3	10
Bulgaria							1					1
Croatia		1	1									2
Denmark		1										1
Estonia						1						1
Finland		2	2									4
France		5	2	1		1	4	1	2		1	17
Germany		3				1			2		4	10
Hungary			1								2	3
Ireland									1			1
Italy		7	5			1	2			1	1	17
Netherlands		3										3
Poland		1									1	2
Portugal			1			1						2
Slovakia			1			1						2
Slovenia						1						1
Spain		1	1			4						6
Sweden		1	1			3	2		1	2	4	14
Turkey			1							1		2
UK	1	1	1	1	1	3	1	2	6	3	5	25
Total	1 (1 %)	28 (22 %)	20 (16 %)	2 (2 %)	1 (1 %)	17 (13 %)	12 (10 %)	3 (2 %)	12 (10 %)	9 (7 %)	21 (17 %)	126

Data from 6 NICUs that did not specify dosage are excluded

^a^Variable dosages, based on child’s condition (took into consideration gestational age, age, birth weight)

Most NICUs who administer fluconazole (85 %) use the intravenous (IV) route only, while 15 % use oral ± IV. NICUs that do not administer fluconazole for fungal treatment often use Liposomal amphotericin B/amphotericin B instead. No factors resulted significant in terms of the NICUs’ choice to use the drug or not.

### Interest in research participation

Almost two-thirds of NICUs (63 %) belong to a research network. Numerous centres reported a willingness to participate in a TINN project evaluating ciprofloxacin (57 %), even though many of these centres (73 %) do not normally use the drug. In general, 85 % of NICUs would be interested in participating in a TINN European multicentre project evaluating medicines in neonates.

## Discussion

### Ciprofloxacin therapy

The results of this survey reveal a heterogeneous situation regarding neonatal sepsis in NICUs throughout Europe, not only concerning drug therapy; for example, the incidence rates of infection vary between NICUs. These could, however, be explained by differences in clinical practice, regardless of birth weight, gestation and disease severity [23, 24]. The most striking result highlighted by this survey is the difference in therapeutic responses to infection in preterm and term neonates in the different NICUs, both between and within countries. The underlying rationales expressed by the NICUs for their choices with respect to the use of these two drugs also vary and reveal uncertainty and a desire for adequate therapeutic data.

Only one in four NICUs uses ciprofloxacin. One of the main concerns expressed by respondents was antibiotic resistance. The use of broad spectrum antibiotics, such as ciprofloxacin, may in fact increase resistance in the unit and may lead to complications in later childhood [8]. Lack of safety and efficacy data in neonates were also issues of concern. In fact, despite their wide use, antibiotics have not been broadly compared for safety and efficacy in the treatment of suspected neonatal sepsis [16, 25]. A retrospective cohort study of premature babies found no effect on linear growth due to ciprofloxacin exposure after 12 months [26]. Furthermore, a recent systematic review [14] confirmed the presence of musculoskeletal adverse events in children, but found that the risk of arthropathy is relatively low and the condition reversible. Further prospective studies on ciprofloxacin safety are needed, however, especially in newborns. Based on this lack of safety and efficacy data, the TINN project aims to conduct a well-designed study to meet this need, which is well-perceived on the part of the NICUs.

No guidelines on the use of ciprofloxacin for sepsis were found. The British national Formulary for Children (BNF-C) [27] recommends other agents for the treatment of neonatal bacteraemia and mentions ciprofloxacin for cases of septicaemia caused by multi-resistant organisms, but it does not provide dosage recommendations. This lack of guidelines is reflected in the data reported in this survey, as dosage ranges vary greatly, both between and within countries.

### Fluconazole therapy

The use of fluconazole for treatment of fungal sepsis is common, even though dosages vary markedly. Fluconazole is suggested as an option in the treatment of invasive candidiasis by the IDSA guidelines, at a dosage of 12 mg/kg daily [13]. The fact that only 16 % of the NICUs reported using this dosage highlights the lack of common, acknowledged guidelines. A measure of additional importance, with respect to other factors, was given to the fact that additional efficacy studies in the perinatal population are needed for this drug. No factors were significant enough, however, to influence the NICUs’ decision to use the drug or not. There was also no correlation between the NICUs’ use of fluconazole for prophylaxis and their use of the drug in treatment [22].

This survey has some limitations, in particular the representativity of the sample. However, as the aim of the study was to describe the prevailing picture of the use of the two drugs in NICUs in Europe, the size of the participating NICUs (even if the actual denominator of those contacted is unknown) and the countries represented can support the reported findings.

## Conclusions

The survey reveals the presence of a great variability in the therapies employed by NICUs, both within and between countries. The differing clinical practices between NICUs, especially in the treatment schemes employed for the treatment of fungal and bacterial infections, need to be addressed at the European level because they underline a lack of evidence-based guidelines harmonizing the different countries [28, 29].

The fact that numerous centres would be interested in participating in research on these drugs, and in research evaluating medicines in neonates in general, demonstrates their desire and recognition of the need to improve therapeutic knowledge in this vulnerable population. These results are extremely positive with respect to TINN’s overall goal because they reveal fertile ground in Europe for researching and implementing homogeneous, internationally acknowledged information and guidance on the treatment of two serious conditions in neonates.

## Appendix 1. Factors taken into consideration in the decision to use or not to use the two drugs

### Rationale for your NICU practice regarding use of ciprofloxacin

Please indicate the importance each of the following factors listed below has on your decision to use or not to use ciprofloxacin in neonates as described above (1: least important, 5: most important):

- Incidence of neonatal sepsis due to multi- drug resistant organisms is/is not high in your NICU
- Ciprofloxacin has a broad bacterial spectrum
- Ciprofloxacin has a good penetration in the cerebrospinal fluid
- Ciprofloxacin is less costly than other antibiotics used in the same indication
- Additional efficacy studies of ciprofloxacin in neonatal bacterial sepsis are needed
- Uncertainty about safety of the use of ciprofloxacin in the newborn is great
- Uncertainty about pharmacokinetics of ciprofloxacin in the newborn is great
- Widespread use could lead to increased bacterial resistance
- Ciprofloxacin should be reserved only for infections with multi-drug resistant microorganisms

### Rationale for fluconazole treatment in your NICU

Please indicate the importance of the following clinical factors listed below has on your decision to prescribe empiric therapy (1 = least important, 5 = most important):

- The agent is less costly compared to other available antifungals
- Statement by Pediatric Societies (AAP) supporting the selection of one antifungal for treatment in neonates is needed
- Uncertainty about pharmacometrics of the other antifungal agents in the newborn is greater than for fluconazole
- Uncertainty about safety of the other antifungal agents in the newborn is greater than for fluconazole
- Additional studies of efficacy in the perinatal population are needed for the other antifungal agents
